# 557. Trends in Remdesivir Treatment Over the Course of the COVID-19 Pandemic

**DOI:** 10.1093/ofid/ofab466.755

**Published:** 2021-12-04

**Authors:** J Xin Liao, Vrishali Lopes, Aisling Caffrey

**Affiliations:** 1 Infectious Diseases Research Program, Providence Veterans Affairs Medical Center, Providence, RI; 2 College of Pharmacy, University of Rhode Island, Kingston, RI, Providence, Rhode Island, PVAMC, Providence, RI; 3 Rhode Island Infectious Diseases Research Program, Providence, RI

## Abstract

**Background:**

Remdesivir is approved for use in the United States for treatment of COVID-19 requiring hospitalization. Real-world data on trends in remdesivir use may elucidate its benefits and place in therapy.

**Methods:**

Hospitalized Veterans with a positive SARS-CoV-2 polymerase chain reaction (PCR) test that were treated with remdesivir at a Veterans Affairs Medical Center from May 2020 to April 2021 were included. Monthly trends in remdesivir treatment, as well as patient characteristics and clinical outcomes among patients treated with remdesivir, were assessed with joinpoint regression to calculate average monthly percent change and corresponding 95% confidence intervals (CI).

**Results:**

A total of 30,333 Veterans were hospitalized with a positive PCR test over the study period, and 13,639 were treated with remdesivir (45%). Throughout the study period, the proportion of Veterans treated with remdesivir increased significantly (4.5% per month, 95% CI 0.5%-8.6%) and median time to remdesivir initiation decreased significantly (12% per month, 95% CI -15.8% to -8.0%). Though demographic characteristics of Veterans treated with remdesivir remained stable, including age, race, and obesity, improvement in clinical outcomes were observed, including median length of hospital stay which decreased by 6.5% per month (95% CI -9.1% to -3.8%), intensive care admissions which decreased by 4.6% per month (95% CI -6.3% to -2.8%) and inpatient mortality which decreased by 6.3% per month (95% CI -9.4% to -3.1%). By April 2021, most patients initiated remdesivir on the day of admission, and the inpatient mortality rate decreased to 7.9% from 19.2% in May 2020.

**Conclusion:**

Over the course of the COVID-19 pandemic, utilization of remdesivir increased while initiation of remdesivir occurred earlier in the hospital admission, with concurrent reductions in length of hospital stay, intensive care admissions, and inpatient mortality.

**Disclosures:**

**Aisling Caffrey, PhD**, **Merck** (Research Grant or Support)**Pfizer** (Research Grant or Support)**Shionogi, Inc** (Research Grant or Support)

